# The antimicrobial and antibiofilm effects of three herbal extracts on *Streptococcus mutans* compared with Chlorhexidine 0.2% (*in vitro* study)

**DOI:** 10.25122/jml-2021-0189

**Published:** 2022-04

**Authors:** Aida Mehdipour, Azita Ehsani, Nasrin Samadi, Marzieh Ehsani, Negar Sharifinejad

**Affiliations:** 1. Department of Pediatric Dentistry, Dental Faculty, Qom University of Medical Sciences, Qom, Iran; 2. Cellular and Molecular Research Center, Qom University of Medical Sciences, Qom, Iran; 3. Department of Oral and Maxillofacial Radiology, Dental Faculty, Hamedan University of Medical Sciences, Hamedan, Iran; 4. Department of Drug and Food Control, School of Pharmacy, Tehran University of Medical Sciences, Tehran, Iran; 5. Dental Faculty, Tehran University of Medical Sciences, Tehran, Iran; 6. Student Research Committee, Qom University of Medical Sciences, Qom, Iran

**Keywords:** *Streptococcus mutans*, *Phlomis bruguieri*, *Marrubium parviflorum*, *Carum copticum*, MIC – Minimum Inhibitory Concentration, DMSO – Dimethyl Sulfoxide, OD – Optical Density, BHI – Brain-heart infusion broth, BHIS – Brain Heart Infusion broth plus sucrose 2%, ATP – Adenosine triphosphate, MDR – Multi drug resistance, PBS – Peripheral Blood Smear

## Abstract

There is a special focus on using natural materials and herbal plants to prevent dental caries. Previous studies showed that some herbal plants have antimicrobial effects on oral pathogens. Thus we investigated the antimicrobial effects of three herbal extracts (*Carum copticum*, *Phlomis bruguieri*, and *Marrubium parviflorum*) on the growth of *Streptococcus mutans*, as the most important bacteria causing dental caries. First, plant methanolic extracts were prepared. Then, to evaluate the antimicrobial activity of the three herbal extracts, the agar well diffusion method and MIC were performed. The biofilm formation was carried out using a broth dilution method with 2% glucose-supplemented BHIS in sterile 96-well microplates. Serial dilutions (50, 25, 12.5, 6.25, 3.12 mg/ml) of extracts were prepared. Next, a 0.5 McFarland Suspension of *S. mutans* was added to wells. The inhibitory effect on biofilm formation was measured by the ELISA reader apparatus. The assay was repeated three times, and the average was calculated as 3. The results were compared with those of Chlorhexidine 0.2%. *Carum copticum* showed a better effect in the agar well diffusion method than others. MIC of the extracts of Carum coptimum, *Phlomis bruguieri*, and *Marrubium parviflorum* were 3.12, 6.25, and 12.5 mg/ml, respectively. Overall, the highest activity belonged to *Carum copticum* extract. For the anti-biofilm effect, the OD values of *Carum copticum* and *Marrubium parviflorum* were significantly different from that of *Phlomis bruguieri*. Although all of the methanolic herbal extracts can inhibit *S. mutans* growth and remove the biofilm, the effect of *Carum copticum* was better than *Phlomis bruguieri* and *Marrubium parviflorum*. Further studies are recommended to indicate how these extracts perform against the bacteria.

## Introduction

Dental caries, gingivitis, and periodontal infections are the most common oral diseases worldwide. Bacteria play a key role in developing dental caries. Two bacteria, *Streptococcus mutans* and *Lactobacillus*, impact dental caries' development and progression more often [1]. Many bacterial species coincide in dental plaques. Oral streptococci, including *S. oralis*, *S. mitis*, and *S. salivarius*, are settled on the surface of the tooth. *S. mutans* form the initial dental plaque and can produce various glucans playing a key role in dental plaque development [2, 3]. In order to assist with mechanical methods, chemical removal of dental plaque by applying disinfectants and mouthwashes is now a common practice [3].

Biofilm is an intricate microbial community. Proteins, lipids, glycopeptides, hydrated polysaccharides, and extracellular DNA make up its structure. It further consists of some organism species, such as fungi, viruses, and bacteria, rooted in an extracellular matrix. The matrix that surrounds the bacteria reduces antimicrobial drug penetration into the biofilm's deeper parts, making it resistant to antimicrobial agents [4].

The most efficient oral rinse for reducing dental plaque germs is Chlorhexidine, which is presently regarded as the gold standard antiplaque agent [5]. It has bactericidal and bacteriostatic characteristics with adequate oral cavity durability and is more efficient against *S. mutans* than *Lactobacillus*. However, using this oral rinse may be limited due to several side effects on the tooth and oral mucosa [6]. The most common side effects of Chlorhexidine are tooth discoloration, change in taste, increased supragingival calculus, allergy, and oral lesions [6–8].

Today, different herbal extracts in treating diseases are frequently reported worldwide. Many secondary metabolites, including alkaloids, terpenoids, glycosides, flavonoids, anthocyanins, lignans, and tannins, are made by plants. Their different parts (leaves, stems, roots, and seeds) store these secondary metabolites [9]. Hence, in recent years, these natural products have been used as adjunctive or alternative therapy to prevent caries [10].

*Carum copticum* (*C. copticum*) or Ajwain is a plant from the Apiaceae family grown in various areas of Asia and Europe, particularly India and Iran [3]. Its therapeutic seed applications include carminative, antimicrobial, amoebiasis expectorant, antiparasitic, antiseptic, antiplatelet-aggregatory, anti lithiasis, and common/acute cold and acute pharyngitis treatment [11].

Some studies reported its oil chemical composition and major components, including γ-terpinene, thymol, and p-cymene [12–14]. One study evaluated the bactericidal activity of *C. copticum* on *Erwinia carotovora*, which can be associated with phenolic compounds, including carvacrol and thymol [15].

Gram-positive bacteria are more susceptible to high thymol doses than gram-negative bacteria. It has been demonstrated that phenolic compounds obstruct cell membranes, alter pH and ion homeostasis, and act as antimicrobial agents [16–19].

The genus *Phlomis* includes 17 species grown in the Iranian provinces of Fars, Gilan, Azerbaijan, Hamadan, Kurdistan, Isfahan, and Mazandaran. Several *Phlomis* species are used to cure respiratory tract problems or heal wounds [20, 21].

*Marrubium* was identified as a tonic, stomachic tonic, antipyretic, and antiseptic in external uses in Iranian-Islamic traditional medicine [6]. It is also useful for nonproductive cough and bronchial asthma. It has been used in different visceral, uterine, and hepatic treatments [22].

There are growing interests in using natural resources, especially medicinal plants, as antimicrobial agents since they do not prompt antibiotic resistance common in artificial antibiotics. These three plants have many traditional uses in Iran, and some studies have shown their antimicrobial effects [17, 23–26]. There are few reports about the antimicrobial effects of these natural products on the main cariogenic microorganism, *Streptococcus* *mutans*. Therefore, the current study investigates the antimicrobial and antibiofilm effects of these extracts on *Streptococcus mutans*.

## Material and Methods

### Preparation of herbal extracts

Aerial parts of *M. parviflorum* were collected near Kadouk in Mazandaran province, and the herbarium number was kept at Islamic Azad University Science and Research Branch, Tehran, Iran (code: 12238-IAUH). Similarly, flowering aerial parts of *Phlomis bruguieri* were collected around Nahavand Hamadan (code: 6911-TEH), and Carum Copticom was bought from the grocery (code: 757-TEH). The voucher specimens were kept at the Herbarium of the Faculty of Pharmacy, Tehran University of Medical science, Iran.

Plant parts were grounded into a fine powder with an electric grinder (Hanchen Plant Grinder, Germany). 100 mg powder of each plant was stored in Erlenmeyer Flask (500 ml) and soaked in 400 ml 80% methanol (Merck, Germany). Whatman No.1 paper was used for filtration. The methanol was evaporated at room temperature under reduced pressure to obtain a methanolic extract. All extracts were finally sterilized via a 0.22 μm pore size membrane and kept in a refrigerator until use.

### Bacterial strains

The Research Organization for Science and Technology (IROST), Iran, provided *Streptococcus mutans* PTCC 1683. The bacteria were cultured on blood agar (Merck, Germany) for 24h at 37°C.

### Antibiogram and MIC

The impact of methanolic extracts on the plants was studied using the agar well diffusion method, as recommended by CLSI [27]. 4g of each extract were dissolved in 2 ml dimethyl sulfoxide (DMSO) with 1% w/v concentration (Merck, Germany), and the stock solutions were obtained. Using 0.5 McFarland standard, the bacterial suspension in sterile normal saline was prepared and cultured on BHI agar media by a sterile cotton swab. Wells were made on the agar plate using the Pasteur pipette (7 mm in diameter with 20 mm distance). Each well was filled with 10 μl of different concentrations of the extracts (0.390, 0.781, 3.1, 6.25, 12.5, 25, 50, 100, and 200 mg/ml). Later, plates were incubated at 37°C for 24h. 

Alcohol-free Chlorhexidine 0.2% and DMSO were applied as positive and negative controls. After the incubation period, inhibition zone diameters were measured in millimeters. The minimum inhibitory concentration (MIC) was assessed as the extracts' lowest concentration inhibiting the bacteria growth on the BHI agar plates. MIC was determined by the inhibition of bacteria. All experiments were performed 3 times for each concentration.

### Anti-pre-formed biofilm activity assay

Biofilm production inhibition by the *Carum copticum*, *Marrubium* *parviflorum*, and *Phlomis bruguieri* extracts was measured based on O'Toole's procedure [28]. Brain-heart infusion broth plus 2% sucrose (BHIS) was the standard biofilm assay medium for *S. mutans* [29]. The bacteria were grown in blood agar overnight; then, 0.5 McFarland standard suspension was obtained, and 20 μL of the bacterial suspension was added per well in a flat-bottom 96-well microtiter plate. Positive control wells contained 200μl bacterial suspension without extracts, while negative controls had only 200μl brain-heart infusion broth plus 2% sucrose (BHIS). For each treatment, 4–8 replicate wells were applied for quantitative assay. For 24h, plates were incubated at 37°C. Following incubation,100 μl of different concentrations (0.390, 0.781, 3.1, 6.25, 12.5, 25, 50, 100, and 200 mg/ml) were added in each well. Next, by turning the plate over, shaking out the liquid, and washing the wells with PBS 3 times, unattached cells were discarded. The crystal violet test was used to assess cell adhesion and biofilm formation [30]. With 200 μl of absolute methanol, attached bacteria were fixed in every well, and the plates were emptied and allowed to dry after fifteen minutes. Then, with 200 μl of 2% solution of crystal violet in water, the wells were stained at room temperature for five minutes. Putting the plate under running tap water, an extra stain was removed. Later, following air drying, the dye bound to the adherent microorganisms was resolubilized with 200 μl of 30% acetic acid in water per well. The absorbance of each well was measured in a microplate reader at 600 nm with a blank of 33 percent acetic acid in water. 

### Statistical analysis

One-way analysis of variance (One-way ANOVA) was used for comparing groups when the distribution of dependent variables was normal. Tukey post hoc test applied for pairwise comparison.

## Results

The antibacterial effects of -00–0.39 mg/mL concentrations of three methanolic herbal extracts and 0.2% Chlorhexidine were evaluated by well diffusion technique. All experiments were performed 3 times for each concentration. The average of inhibition zones for each concentration is reported in Table 1.

**Table 1.** The zone of inhibition for experimental herbal extracts against Streptococcus mutans.

**Herbal extracts**	**Extract Concentrations(mg/ml)**								
	**200**	**100**	**50**	**25**	**12.5**	**6.25**	**3.125**	**0.78**	**0.39**
***Carum copticum***	24.33	20.66	19	15.16	12.33	9.66	7.66	0	0
***Marrubium parviflorum***	17.33	15.33	13.66	12.33	9.33	8.66	0	0	0
***Phlomis bruguieri***	16.33	12.33	10.33	8.66	8.33	0	0	0	0

No bacteria growth was observed in low concentrations of 0.78 mg/ml. *Carum copticum* was more effective against *S. mutans* than *Marrubium parviflorum* and *Phlomis bruguieri*. 

Minimum inhibitory concentrations (MIC) for *Carum* *copticum*, *Marrubium parviflorum*, and *Phlomis bruguieri* were 3.125, 6.25, and 12.5 mg/ml, respectively (Table 2). Chlorhexidine, as the positive control, showed a 30 mm inhibition zone, and DMSO, as the negative control, did not have any effect on *Streptococcus* *mutans*.

**Table 2.** The minimum inhibitory concentration of herbal extracts against Streptococcus mutans.

**Herbal extracts**	**Minimal inhibitory concentration (MIC) (mg/ml)**
***Carum copticum***	3.125
***Marrubium parviflorum***	6.25
***Phlomis bruguieri***	12.5

The antibacterial activities of Chlorhexidine mouthwash were significantly better than all three herbal extracts. Overall, the findings indicated that *Carum copticum* extract was more effective than the two other extracts. 

The optical density (OD) values are positively correlated with the density of bacteria aggregated as a biofilm on plate walls. Therefore, lower OD values indicate higher efficacy in inhibiting biofilm formation and vice versa.

The OD value for *Carum copticum* was not significantly different from that of *Marrubium parviflorum* at a given concentration (P=.273). However, the OD values for *Carum copticum* and *Marrubium* *parviflorum* were significantly different from that of *Phlomis bruguieri* (P=.021 and .006, respectively) (Tables 3 and 4). Mean and standard deviation of OD in extracts, Chlorhexidine, negative and positive control are mentioned in Table 5.

**Table 3.** Comparison of OD between extracts.

**Extract**	**Extract**	**Average difference**	**P-value**
***Carum copticum***	*Marrubium parviflorum*	-0.301	0.273
	*Phlomis bruguieri*	-0.045	0.021
***Marrubium parviflorum***	Phlomis bruguieri	-0.015	0.006

**Table 4.** Comparison of OD between Chlorhexidine and other extracts.

	Extracts	Average difference	P-value
***Chlorhexidine***	Carum copticum	-0.064	0.013
	*Marrubium parviflorum*	-0.094	0.037
	*Phlomis bruguieri*	-0.110	0.009
	*Positive control*	-0.191	0.000
	*Negative control*	0.201	0.000

**Table 5.** Mean and standard deviation of OD in extracts, Chlorhexidine, and negative and positive control.

		**OD in different concentration (mg/ml)**								
**200** **(mg/ml)**	**100** **(mg/ml)**	**50** **(mg/ml)**	**25** (mg/ml)	**12.5** **(mg/ml)**	**6.25** **(mg/ml)**	**3.12** **(mg/ml)**	**1.56** **(mg/ml)**	**0.78** **(mg/ml)**
***Carum copticum***	Mean	0.309	0.318	0.326	0.363	0.379	0.384	0.402	0.430	0.468
	Standard deviation	0.0055	0.0037	0.0051	0.0035	0.003	0.0035	0.0047	0.004	0.004
***Marrubium parviflorum***	Mean	0.331	0.349	0.369	0.388	0.391	0.410	0.422	0.483	0.488
	Standard deviation	0.002	0.0025	0.003	0.003	0.0041	0.0036	0.0035	0.005	0.004
***Phlomis bruguieri***	Mean	0.331	0.358	0.381	0.411	0.419	0.423	0.450	0.495	0.493
	Standard deviation	0.002	0.002	0.002	0.002	0.002	0.002	0.0025	0.002	0.0037
***Chlorhexidine***	Mean	0.308								
	Standard deviation	0.0045								
***Positive control***	Mean	0.499								
	Standard deviation	0.0051								
***Negative control***	Mean	0.106								
	Standard deviation	0.004								

**Figure 1.**
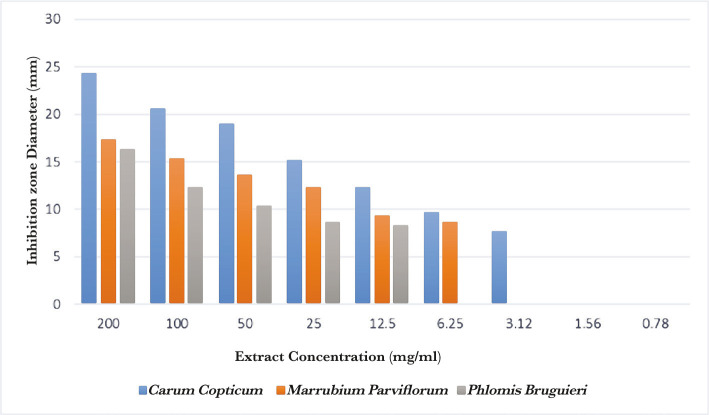
Comparison between inhibition zone diameters of three extracts in different extract concentrations.

**Figure 2.**
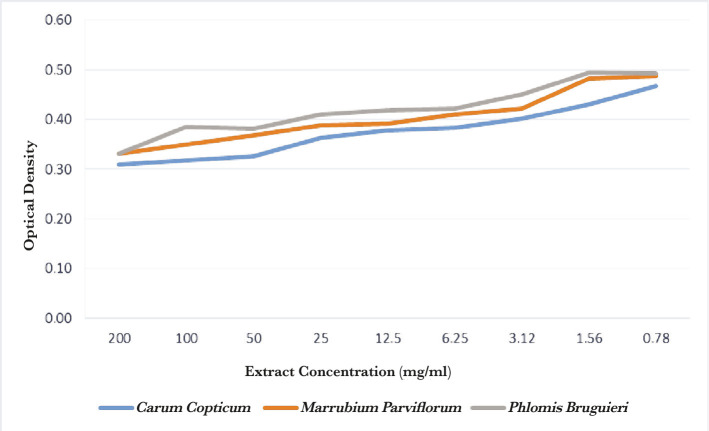
Correlation between different extract concentrations and OD.

The correlations of extract concentrations with Inhibition zone diameter and OD are shown in Figures 1 and 2, respectively.

## Discussion

This study evaluated the impact of three herbal extracts with various concentrations on the most significant bacterial strain in dental pathologies. The polyphenol toxicity effectiveness on microbes can be associated with the inhibition of hydrolytic enzymes (carbohydrases and proteases) or other interactions to cell envelope transport proteins, inactive microbial bonds, as well as general interactions with carbohydrates [31].

In the current research, since Chlorhexidine gluconate has a significant antibacterial impact on *S. mutans*, it was applied as a positive and efficient agent against *S. mutans* [32]. Chlorhexidine is most effective on gram-positive bacteria; however, it also affects gram-negative bacteria, fungi anaerobes, and several enveloped viruses. It is not effective on bacterial spores. Every patient is advised by their dentist to use this agent. Staphylococci species have decreased susceptibility to Chlorhexidine [33].

When used alone or in combination, natural herbs have been scientifically proven to be safe against a variety of oral health issues such as bleeding gums, halitosis, mouth ulcers, and decay [34]. In many countries, many plant products have been successfully combined into dentifrice or mouthwash [35].

The different concentrations of Carum coptimum, *Phlomis bruguieri*, and *Marrubium parviflorum* extracts (0.390, 0.781, 3.125, 6.25, 12.5, 25, 50, 100, and200 mg/mL) were less effective compared to 0.2% Chlorhexidine gluconate. The essential oil of *C. copticum* contains a significant amount of thymol, which contributes to its antimicrobial activity. Its mechanism appears to be primarily associated with the damaging effects on both the cellular cytoplasmic membrane (puncture) and ATP production [36].

The antibacterial impacts of 0.78–200 mg/mL concentrations of three methanolic herbal extracts and 0.2%Chlorhexidine were examined using the well-diffusion technique. According to reports, the high concentrations of thymol and carvacrol usually inhibit gram-positive more than gram-negative bacteria [37, 38].

The ether fraction of C.copticum has a better antifungal, and antibacterial effect than other fractions on multidrug-resistant (MDR) strains of *Candida albicans*, *Candida tropicalis*, *Candida krusei*, *Escherichia coli*, *Candida glabrata*, as well as standard strains of *Streptococcus mutans* and *Streptococcus bovis* [39].

Diterpenes, caffeic acid derivatives, sterols, and flavonoids are abundant in *Marrubium* species [35]. Another study (in Nevsehir, Turkey) found that the major chemical compounds in *M. parviflorum* essential oil are hexadecanoic acid (15.4%), germacrene D (11.1%), and caryophyllene (10%) [34, 40].

Some *Phlomis* species have been applied in herbal medicine, *e.g.*, in the respiratory tract or externally for wound treatment [20]. The genus *Phlomis* plants have been reported to involve flavonoid glycosides, iridoid glucosides, diterpene glycosyl esters, phenylethanoid glycosides and nortriterpenes [41, 42].

The methanol extracts of the dried flowering aerial parts of *P. herba-venti*, *Phlomis bruguieri*, and *P. olivieri*, showed concentration-dependent antibacterial activity against all bacteria tested. Such extracts were more effective on gram-positive microorganisms (*Staphylococcus aureus* and *Streptococcus sanguis*).

They were most efficient on *Streptococcus sanguis*. However, they did not exhibit antifungal effects [43]. The total extract of *P. olivieri* showed antinociceptive impact in the visceral writhing mice model [44].

This study also investigated the antibiofilm activity of these extracts via the microtitre plate method. Chlorhexidine had the lowest OD compared with the three extracts at a given concentration. Hence, it demonstrated the strongest antibiofilm effect. The highest antibiofilm activity was observed in *Carum copticum* and *Marrubium parviflorum*. The results regarding *Carum copticum* are consistent with the findings of Khan and colleagues [45]. They studied the antibiofilm and antiadhesive effects of propyl-octa-hydro naphthalene in *C. copticum* on *S. mutans*, showing its antibiofilm activity [39]. We also showed a potent antibiofilm activity of *Marrubium parviflorum*. In a study of antibiofilm and antioxidant activities of methanolic extract of *Marrubium deserti* against a number of bacterial strains, Chemsa *et al.* attributed the plant antibiofilm activity to flavonoids and phenol compounds [46]. The antibiofilm activity of *Phlomis bruguieri* was not studied before, although we could show a modest effect.

Phenolic compounds are demonstrated to show a variety of biological activities, including antibacterial, antioxidant, and anti-inflammatory properties. Plants such as *M. parviflorum* extracts contain high amounts of phenolic compounds. Flavonoids are the most common group of plant phenolic ingredients characterized by a benzo-y-pyrone structure. Flavonols are a class of flavonoids that have the 3-hydroxy flavone backbone. Their diversity stems from the various positions of the phenolic -OH groups. Flavonols exist in methanol extracts of *M. parviflorum* [47].

The major compounds of *C. copticum* are thymol (and its precursors), cymene, and terpinene [48].

It has been demonstrated that thymol and its precursors, cymene and terpinene, [49, 50] have strong antibacterial effects. Thymol induces its antibacterial effect by perturbing the lipid fraction of the microorganism plasma membrane, causing changes in the membrane permeability and leakage of intracellular materials [51].

Although terpinene was the second main compound identified, no strong antimicrobial effect was demonstrated from its gamma isomer [21].

P-cymene is another major compound identified as a hydrophobic molecule that causes swelling of the cytoplasmic membrane [52]. When applied alone, it is not a strong antimicrobial element [53, 54]. However, in combination with other phenolic materials such as carvacrol, it has demonstrated a strong antibacterial activity by injecting cymene in the lipid bilayer of bacteria [55].

*Phlomis* species have been represented to have antibacterial, antidiabetic, antinociceptive [10], antimutagenic, and anti-inflammatory properties. These plants contain different classes of glycosides involving diterpenoids, iridoids, phenylethanoids, phenylpropanoids, and flavonoids. Many relations between the properties of these plants and some compounds have been demonstrated. For example, many phenylpropanoids showed significant effects such as cytotoxic, cytostatic, anti-inflammatory, immunosuppressant, and antimicrobial characteristics.

Also, saturated and unsaturated fatty acids are detected in these plants. Palmitic acid and nonahexacontanoic acid were two saturated fatty acids identified in *P. bruguieri* [56].

## Conclusion

This study showed *Carum copticum*, Marsupium parviflorum, and *Phlomis bruguieri* extracts to have antibacterial activities on *Streptococcus mutans*. This effect was dependent on their concentration. Based on the research results, the extracts of the three plants possess antibacterial and antibiofilm activities against *S. mutans*, with *Carum copticum* and *Phlomis bruguieri* exhibiting the highest and lowest activities, respectively. The antibiofilm activity of the three extracts was lower than the common 0.2% Chlorhexidine mouthwash. Since medicinal plants are abundant in Iran and pose fewer side effects than chemicals, research to identify candidate plants in dental and oral care is essential. Further, in vivo studies are necessary on the antibiofilm and antibacterial activities of such extracts. Accordingly, further research is recommended to estimate in vivo effects of the extracts.

## Acknowledgments

### Conflict of interest

The authors declare no conflict of interest.

### Ethical approval

This study was approved by the Ethics Committee of Qom University of Medical Science, Qom, Iran (approval ID: IR.MUQ.Rec.1395.015)

### Data availability

The datasets generated and/or analyzed during the current study are not publicly available due to subject matter specialization but are available from the corresponding author on reasonable request.

### Funding

The research was funded by the Vice for Research and Technology of Qom University of Medical Sciences, Qom, Iran.

### Authorship

AM and AE developed and supervised the work. AE and ME performed the experiments. AM and AE contributed to writing the original draft. ME and NSh contributed to editing the manuscript. NS contributed to data interpretation. All authors reviewed the manuscript. All authors read and approved the final manuscript.
